# Normal high-sensitivity cardiac troponin for ruling-out inpatient mortality in acute COVID-19

**DOI:** 10.1371/journal.pone.0284523

**Published:** 2023-04-21

**Authors:** Alexander Liu, Robert Hammond, Kenneth Chan, Chukwugozie Chukwuenweniwe, Rebecca Johnson, Duaa Khair, Eleanor Duck, Oluwaseun Olubodun, Kristian Barwick, Winston Banya, James Stirrup, Peter D. Donnelly, Juan Carlos Kaski, Anthony R. M. Coates

**Affiliations:** 1 University of St Andrews School of Medicine, St Andrews, United Kingdom; 2 Royal Berkshire NHS Foundation Trust, Reading, United Kingdom; 3 Royal Brompton Hospital, London, United Kingdom; 4 Molecular and Clinical Sciences Research Institute, St George’s University of London, London, United Kingdom; 5 St George’s, University of London, London, United Kingdom; Keio University School of Medicine, JAPAN

## Abstract

**Introduction:**

Assessment of inpatient mortality risk in COVID-19 patients is important for guiding clinical decision-making. High sensitivity cardiac troponin T (hs-cTnT) is a biomarker of cardiac injury associated with a worse prognosis in COVID-19. We explored how hs-cTnT could potentially be used in clinical practice for ruling in and ruling out mortality in COVID-19.

**Method:**

We tested the diagnostic value of hs-cTnT in laboratory-confirmed COVID-19 patients (≥18 years old) admitted to the Royal Berkshire Hospital (UK) between 1^st^ March and 10^th^ May 2020. A normal hs-cTnT was defined as a value within the 99^th^ percentile of healthy individuals (≤14 ng/L), and an elevated hs-cTnT was defined as >14 ng/L. Adverse clinical outcome was defined as inpatient mortality related to COVID-19.

**Results:**

A total of 191 COVID-19 patients (62% male; age 66±16 years) had hs-cTnT measured on admission. Of these patients, 124 (65%) had elevated hs-cTnT and 67 (35%) had normal hs-cTnT. On a group level, patients with elevated hs-cTnT had worse inpatient survival (p = 0.0014; Kaplan-Meier analysis) and higher risk of inpatient mortality (HR 5.84 [95% CI 1.29–26.4]; p = 0.02; Cox multivariate regression) compared to patients with normal hs-cTnT. On a per-patient level, a normal hs-cTnT had a negative predictive value of 94% (95% CI: 85–98%) for ruling out mortality, whilst an elevated hs-cTnT had a low positive predictive value of 38% (95% CI: 39–47%) for ruling in mortality.

**Conclusions:**

In this study cohort of COVID-19 patients, the potential clinical utility of hs-cTnT appears to rest in ruling out inpatient mortality. This finding, if prospectively validated in a larger study, may allow hs-cTnT to become an important biomarker to facilitate admission-avoidance and early safe discharge.

## Introduction

In the COVID-19 pandemic, reducing the rates of hospital admissions and the duration of inpatient stay remain two of the most important clinical priorities [1–4]. On the medical frontline, identifying patients at a low risk for inpatient mortality can offer more confidence in clinical decision-making involving admission-avoidance and/or early discharge [1–4].

In COVID-19 patients, cardiac injury is associated with an increased mortality risk [5–12]. Assessment of cardiac injury therefore forms an important aspect of clinical risk stratification [8]. High sensitivity cardiac troponin T is a widely available biomarker of cardiac injury [13]. Cardiac troponin is also associated with underlying comorbidities and mortality prediction [14–18]. Despite strong evidence suggesting that COVID-19 patients with elevated cardiac troponin levels exhibit higher mortality risk than those with normal levels [18], cardiac troponin is still not being used in clinical practice for risk-stratifying COVID-19 patients. The reasons behind this lack of clinical translation are unclear. In this study, we sought to examine the individual diagnostic potential of cardiac troponin for predicting inpatient mortality, in order to identify the manner in which it could be used for risk-stratification in COVID-19. We hypothesised that high sensitivity cardiac troponin T can accurately predict inpatient mortality on a diagnostic level in acute COVID-19 patients.

## Materials and methods

### Study subjects

We retrospectively analysed all consecutive patients who had both laboratory-confirmed COVID-19 and high sensitivity cardiac troponin T measurement (Roche Elecsys ®) admitted to the Royal Berkshire National Health Services (NHS) Foundation Trust (Reading, UK) between 1^st^ March 2020 and 10^th^ May 2020. COVID-19 was diagnosed by real‐time reverse transcriptase polymerase chain reaction (rt-PCR) testing for SARS-CoV-2 from nasopharyngeal swabs, as previously described [10,19,20]. The Trust serves over 500,000 people within its catchment area [21]. Hs-cTnT tests were requested by the treating physicians as part of routine clinical care. Patients under the age of 18 were excluded.

### Data collection

From the electronic medical records, we collected data on demographics status (such as age, gender, body mass index), clinical presentation (such as symptomology, co-morbidities, smoking history and medications), laboratory tests, chest x-rays and electrocardiogram (ECG) data. In brief, a standardised data collection protocol and a spreadsheet template were produced and circulated to all investigators involved in raw data collection. The investigators were each allocated a proportion of patients and were asked to collect ten initial sample cases. These were validated for accuracy by another observer against the medical records before the investigators completed the remaining data collection. To ensure utmost accuracy, the study data were validated again, referenced to electronic medical records, by two observers before any data analysis was performed.

### Ethics statement

This study was granted COVID-19 Fast-Track Approval by the Health Research Authority (HRA) and Health and Care Research Wales (HCRW), UK. IRAS project ID: 287103. The data collectors required access to patient identifiable information during data collection. After all the data were collected and checked, the data were fully anonymised to be free from any patient identifiable information. This study involves the retrospective analysis of already collected anonymised data, no informed consent was required.

### Endpoint definitions

Hs-cTnT assays were analysed using the Roche Cobas e801 analyser (Roche Diagnostics, Mannheim, Germany) with an analytical range of 3–10,000 ng/L, a 99th percentile upper reference limit of 14 ng/L and limit of quantitation of 10% CV (intermediate) at 5.48 ng/L. Patients were categorised as having either normal or elevated cardiac troponin based on a cut-off derived from healthy individual values, as previously described [22,23]. A normal troponin level was defined as a value within 99^th^ percentile of healthy individuals (≤14 ng/L) [22,23]. An elevated troponin level was defined as a value greater than 99^th^ percentile of healthy individuals (>14 ng/L) [22,23]. Adverse clinical outcome was defined as inpatient mortality related to acute COVID-19.

### Statistical analysis

Continuous variables were checked for normality using the Kolmogorov-Smirnov test [24]. Parametric data were expressed as mean (SD) [5,8,25]. Non-parametric data were expressed as median (inter-quartile range [IQR]) [5,8,25]. Comparisons between parametric data were performed using the unpaired Student’s t-test [25]. Comparisons between non-parametric data were performed using the Mann-Whitney test [25]. Categorical data were compared using the Chi-square test or the Fisher Exact test [25,26]. Kaplan-Meier curves were used to assess inpatient survival in COVID-19 patients [27]. The Logrank rest was used to compared inpatient survival in COVID-19 patients with normal vs elevated troponin. Multi-variate Cox proportional-hazard regression analysis was used to assess inpatient mortality risk for troponin and other common risk factors; hazard ratios (HR) were presented with 95% confidence intervals (CI) [28]. Receiver-Operating Characteristics (ROC) analysis was performed to assess the diagnostic performance of troponin for predicting inpatient mortality in COVID-19 patients; area under the ROC curve was presented with 95% CI [29]. Statistical significance was defined as p<0.05. The statistical analysis was first performed by AL (using MedCalc; Version 12.7.8.0), which was then independently validation by WB who is an expert medical statistician (using Stata; Basic Edition version 17.0, Statacorp LLC, Texas USA).

## Results

### Patient characteristics

Baseline patient characteristics are summarised in Table 1. In total, 191 COVID-19 patients (mean age 65.8 ± 16.3 years; 62.3% male) had high sensitivity cardiac troponin T measured on admission. Compared to patients with normal troponin, patients with elevated troponin had lower BMI and a lower proportion of patients from Black Asian and Minority Ethnics (BAME). Patients with elevated troponin also had a lower burden of symptoms such as chest pain, dyspnoea, cough and fever (Table 1).

**Table 1:** Baseline patient characteristics.

	All Patients (n = 191)	Hs-cTnT (≤14 ng/L) (n = 67)	Hs-cTnT (>14 ng/L) (n = 124)	P value
Age (years)	65.8 ± 16.3	53.6 ± 13.6	72.4 ± 13.7	<0.0001
Male (%)	119 (62.3)	35 (52.2)	84 (67.7)	0.035
BMI (kg/m^2^)	27.5 (23.8–31.9)	29.8 (25.1–33.2)	26.4 (22.4–30.3)	0.012
BAME	48/174 (27.6)	24/60 (40.0)	24/114 (21.1)	0.008
Symptoms				
Chest pain	40/190 (21.1)	21/66 (31.8)	19 (15.3)	0.015
Dyspnoea	128/190 (67.4)	54/66 (81.8)	74 (59.7)	0.002
Palpitations	5/190 (2.6)	2/66 (3.0)	3 (2.4)	1.000
Fatigue	41/190 (21.6)	13/66 (19.7)	28 (22.6)	0.65
Cough	116/190 (61.1)	56/66 (84.9)	60 (48.4)	<0.0001
Fever	101/190 (53.2)	50/66 (75.8)	51 (41.1)	<0.0001
Diarrhoea	29/190 (15.3)	12/66 (18.2)	17 (13.7)	0.41
Anosmia / Ageusia +	8/119 (6.7)	4/38 (10.5)	4/81 (4.9)	0.265
Comorbidities				
IHD	34/188 (18.1)	3/65 (4.6)	31/123 (25.2)	0.0003
Heart failure	22/188 (11.7)	0 (0)	22/123 (17.9)	<0.0001
Hypertension	84/189 (44.4)	13/65 (20.0)	71 (57.3)	<0.0001
Diabetes	60/188 (31.9)	9/64 (14.1)	51 (41.1)	<0.0001
Dyslipidaemia	19/188 (10.1)	4/64 (6.3)	15 (12.1)	0.213
Current Smoker	10/179 (5.6)	3/60 (5.0)	7/119 (5.9)	1.000
Ex-Smoker	45/179 (25.1)	10/60 (16.7)	35/119 (29.4)	0.06
AF	25/189 (13.2)	2/65 (3.1)	23 (18.6)	0.003
CKD	44/189 (23.3)	1/65 (1.5)	43 (34.7)	<0.0001
COPD	17/189 (9.0)	1/65 (1.5)	16 (12.9)	0.009
Asthma	25/189 (13.2)	12/65 (18.5)	13 (10.5)	0.12
Cerebrovascular disease	18/189 (9.5)	1/65 (1.5)	17 (13.7)	0.007
Dementia	17/189 (9)	2/65 (3.1)	15 (12.1)	0.04
Cancer	12/189 (6.4)	3/65 (4.6)	9 (7.3)	0.55
Medications				
ACEi / ARB	48/186 (25.8)	10/65 (15.4)	38/121 (31.4)	0.017
Beta-Blockers	50/186 (26.9)	6/65 (9.2)	44/121 (36.4)	<0.0001
CCB	46/186 (24.7)	6/65 (9.2)	40/121 (33.1)	<0.0001
Aspirin/Clopidogrel	28/186 (15.1)	5/65 (7.7)	23/121 (19.0)	0.04
Digoxin +	3/186 (1.6)	0 (0)	3/121 (2.5)	0.553
Warfarin +	8/186 (4.3)	0 (0)	8/121 (6.6)	0.052
DOAC	25/186 (13.4)	3/65 (4.6)	22/121 (18.2)	0.01
MRA +	6/186 (3.2)	0 (0)	6/121 (5.0)	0.093
Nitrates +	5/186 (2.7)	0 (0)	5/121 (4.1)	0.164
Statins	66/186 (35.5)	15/65 (23.1)	51/121 (42.2)	0.01

Hs-cTnT: High sensitivity cardiac troponin T; BMI: Body mass index; BAME: Black, Asian and minority ethnic; IHD: Ischaemic heart disease; AF: Atrial fibrillation; CKD: Chronic kidney disease; COPD: Chronic obstructive pulmonary disease; ACEi: Angiotensin converting enzyme inhibitor; ARB: Angiotensin receptor blocker; CCB: Calcium channel blocker; DOAC: Direct oral anticoagulant; MRA: Mineralocorticoid receptor agonist. + denotes categorical data comparisons performed by the Fishers exact test; remaining categorical data comparisons were performed using the Chi-squared test.

Patients with elevated troponin had a higher prevalence of co-morbidities, including ischaemic heart disease (IHD), hypertension, heart failure, diabetes mellitus (DM) and atrial fibrillation (AF). Chronic kidney disease (CKD), chronic obstructive pulmonary disease (COPD), cerebrovascular disease and dementia were also more frequently seen in patients with elevated troponin (Table 1). A greater proportion of patients with elevated troponin took regular cardiac medications (Table 1).

### Patient observation, test results and complications

On admission, patients with elevated troponin had similar temperature, systolic blood pressure, respiratory rate and prevalence of hypoxia, compared to patients with normal troponin (Table 2). The prevalence of chest radiograph abnormalities was also similar between the two groups. Patients with elevated troponin also had wider QRS complexes and QTc intervals, as well as lower haemoglobin, haematocrit, lymphocyte count, creatinine and d-dimer (Table 2).

**Table 2:** Patient observations, investigation results and complications.

	All Patients (n = 191)	Hs-cTnT (≤14 ng/L) (n = 67)	Hs-cTnT (>14 ng/L) (n = 124)	P value
Admission Observation				
Temperature (°C)	37.3 ± 1.2	37.3 ± 1.3	37.3 ± 1.3)	0.97
SBP (mmHg)	127 (114–143)	129 (118, 140)	125 (112–144)	0.244
DBP (mmHg)	75 ± 14	78 ± 12	74 ± 14	0.06
Respiratory Rate (/min)	22 (19–28)	22 (19–28)	23 (19–28)	0.289
Significant Hypoxia*	45/185 (24.3)	11/66 (16.7)	34/119 (28.6)	0.07
Chest radiograph				
Consolidation	41/185 (22.2)	16/66 (24.2)	25/119 (21.0)	0.612
Opacification	73 (38)	28 (42)	45 (36)	0.533
Atelectasis	18/185 (9.7)	4/66 (6.1)	14/119 (11.8)	0.210
Pleural Effusion	10/185 (5.4)	0 (0)	10/119 (8.4)	0.016
ECG				
Heart rate (bpm)	88 (75–102)	88 (77–101)	90 (75–104)	0.590
PR interval (ms)	155 (140–174)	155 (138–174)	155 (141–176)	0.456
QRS duration (ms)	97 (87–108)	96 (86–102)	100 (88–114)	0.011
QTc duration (ms)	410.8 ± 38.5	396.8 ± 37.1	418.1 ± 37.4	0.0004
Laboratory Results				
Haemoglobin (g/L)	128 (113–144)	141 (124–150)	123 (107–139)	<0.0001
Haematocrit	0.39 (0.35–0.44)	0.41 (0.38–0.44)	0.37 (0.34–0.42)	0.0001
WCC (10^9^/L)	7.6 (5.5–10.3)	7.6 (5.1–9.8)	7.7 (5.6–11.5)	0.238
Platelet Count (10^9^/L)	228 (178–299)	225 (181–293)	231 9177–302)	0.97
Lymphocyte Count (×10^9^/L)	0.90 (0.69–1.29)	1.08 (0.89–1.58)	0.80 (0.54–1.14)	<0.0001
Sodium (mmol/L)	138 (135–140)	138 (134–140)	138 (135–141)	0.31
Potassium (mmol/L)	4.2 (3.9–4.5)	4.1 (3.9–4.3)	4.2 (3.8–4.6)	0.374
Creatinine (μmol/L)	86 (67–137)	71 (62–85)	105 (77–185)	<0.0001
Ferritin (μg/L)	753 (297–1493)	775 (161–1409)	739 (394–1657)	0.295
CRP (mg/L)	115 (45–212)	92 (28–207)	131 (55–229)	0.090
D-Dimer (ng/ml)	1104 (663–3037)	885 (550–1377)	1605 (800–3676)	0.004
Creatine Kinase (U/L)	100 (64–241)	102 (56–240)	96 (71–271)	0.566
Complications				
NIV requirement	47 (24.6)	13 (19.4)	34 (27.4)	0.220
ICU admission	33 (17.3)	15 (22.4)	18 (14.5)	0.170
Intubation	15 (7.9)	6 (9.0)	9 (7.3)	0.677
Inpatient Mortality	51 (26.7)	4 (6.0)	47 (37.9)	<0.0001

Hs-cTnT: High sensitivity cardiac troponin T; SBP: Systolic blood pressure; DBP: Diastolic blood pressure; ECG: Electrocardiogram; WCC: White cell count; CRP: C-reactive protein; NIV: Non-invasive ventilation; ICU: Intensive care unit. *FiO_2_ requirement >50% [30].

Of the 191 patients in the study, 47 (24.6%) required non-invasive ventilation (NIV), 33 (17.3%) were admitted to the intensive care unit (ICU), 15 (7.9%) required intubation and 51 (26.7%) suffered inpatient mortality. The prevalence of inpatient mortality was significantly greater in patients with elevated troponin compared to patients with normal troponin (37.9% vs 6.0%, p<0.0001). Patients with elevated troponin and patients with normal troponin had similar rates of NIV requirement (27.4% vs 19.4%, p = 0.220), ICU admissions (14.5% vs 22.4%, p = 0.170) and intubation (7.3% vs 9.0%, p = 0.677; Table 2).

### Survival analysis

**Fig 1:**
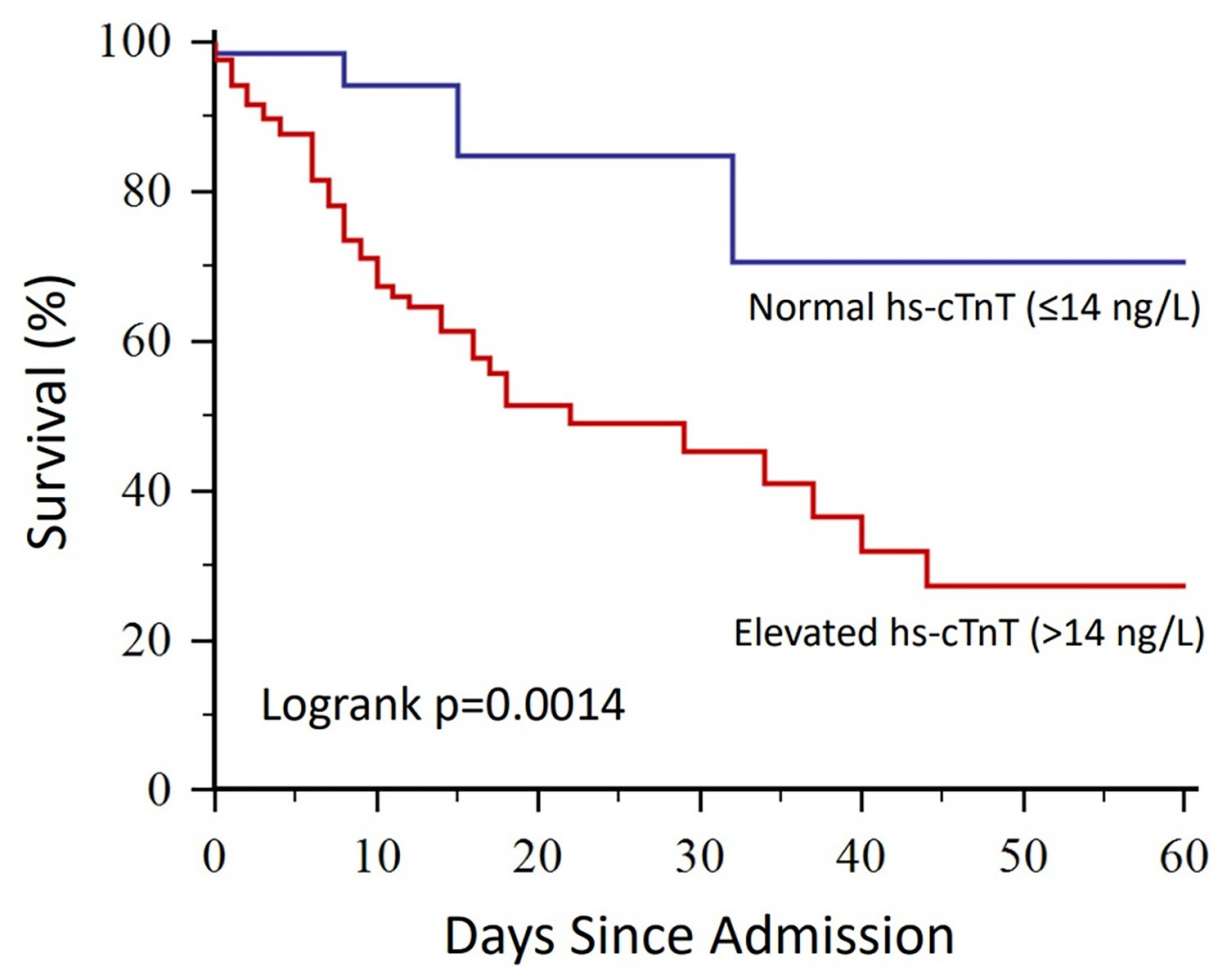
Kaplan Meier curves showing inpatient survival in coronavirus disease 19 (COVID-19) patients with normal versus elevated high sensitivity cardiac troponin T (hs-cTnT).

**Fig 2:**
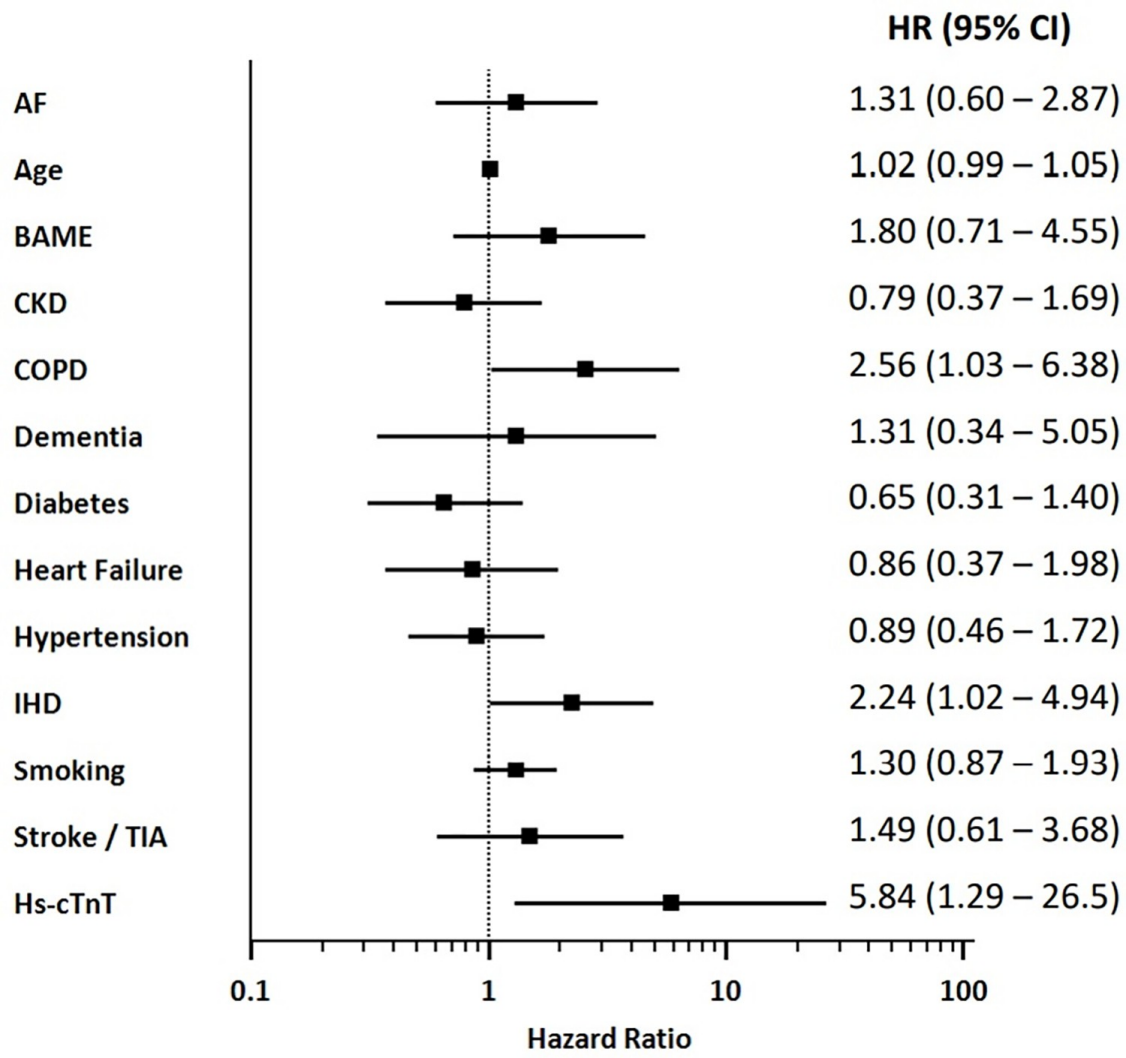
A Forrest plot showing the hazard ratios (HR) of multiple variables for inpatient mortality, as calculated using the multi-variate Cox proportional-hazard regression analysis. HR are displayed with 95% confidence intervals (CI). AF: Atrial fibrillation; BAME: Black, Asian and minority ethnic; CKD: Chronic kidney disease; COPD: Chronic obstructive pulmonary disease; IHD: Ischaemic heart disease; TIA: Transient ischaemic attack; Hs-cTnT: High sensitivity cardiac troponin T.

On Kaplan Meier analysis, COVID-19 patients with elevated troponin had significantly worse inpatient survival than patients with normal troponin (p = 0.0014, logrank test; Fig 1). Cox multivariate regression analysis was performed with a range of clinically important variables including age, BAME status, CKD, chronic COPD, diabetes, stroke / TIA, smoking status, heart failure, hypertension, IHD, AF, dementia and troponin. Patients with elevated troponin had a significantly higher risk of inpatient mortality than patients with normal troponin (HR 5.84, 95% CI 1.29–26.5, p = 0.023; Fig 2), independent of the other variables in the analysis. In this study, other independent predictors of mortality in COVID-19 patients were IHD (HR 2.24, 95% CI 1.02–4.94, p = 0.047) and COPD (HR 2.56, 95% CI 1.03–6.38], p = 0.045; Fig 2). Univariate cox regression values are shown in Table 3 for reference.

**Table 3:** Univariate cox regression analysis of predictors of mortality in COVID-19.

	Hazard Ratio (95% CI)	P value
AF	1.86 (0.97–3.57)	0.06
Age	1.04 (1.02–1.06)	<0.0001
BAME	1.21 (0.63–2.31)	0.56
CKD	1.65 (0.93–2.91)	0.09
COPD	2.71 (1.35–5.44)	0.005
Dementia	1.26 (0.50–3.17)	0.63
Diabetes mellitus	1.02 (0.58–1.81)	0.94
Heart failure	2.02 (1.03–3.96)	0.04
Hypertension	0.96 (0.55–1.68)	0.90
IHD	2.42 (1.34–4.34)	0.003
Smoking (ex- or current)	1.37 (1.01–1.87)	0.04
Cerebrovascular disease	2.07 (0.97–4.42)	0.06
Hs-cTnT	4.51 (1.62–12.53)	0.004

AF: Atrial fibrillation; BAME: Black, asian and minority ethnic; CI: Confidence interval; CKD: Chronic kidney disease; COPD: Chronic obstructive pulmonary disease; Hs-cTnT: High sensitivity cardiac troponin T; IHD: Ischaemic heart disease.

### Predictive value of high sensitivity cardiac troponin T for inpatient mortality

**Fig 3:**
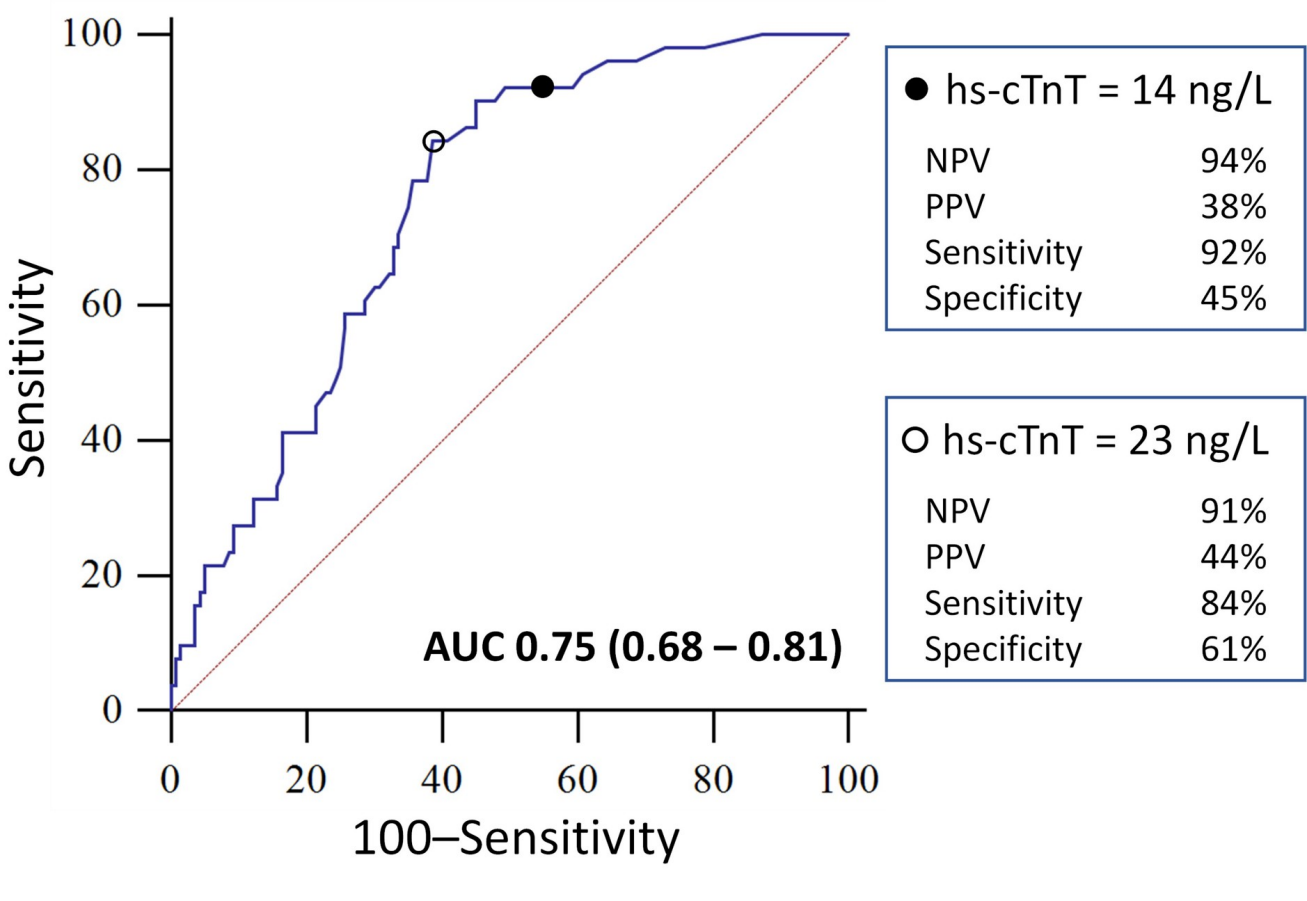
Receiver-Operating Characteristic (ROC) curve of the diagnostic performance of high sensitivity cardiac troponin T (hs-cTnT) for predicting inpatient mortality in patients with coronavirus disease 19 (COVID-19). A total of 191 patients were included in the ROC analysis. The hs-cTnT threshold of 14 ng/L was based on the 99^th^ percentile of healthy individuals (denoted by the filled dot in the top panel). The hs-cTnT threshold of 23 ng/L was based on the Youden point of the ROC curve (denoted by the empty dot in the bottom panel). AUC denotes area under the ROC curve; values in brackets denote the 95% confidence interval for the AUC with p value <0.0001.

For predicting inpatient mortality in COVID-19 patients on ROC analysis (AUC = 0.75, 95% CI: 0.68–0.81; Fig 3), a normal troponin (<14 ng/L) achieved a sensitivity of 92% (95% CI: 81–98%), a specificity of 45% (95% CI: 37–54%), a positive predictive value (PPV) of 38% (95% CI: 29–47%) and a negative predictive value (NPV) of 94% (95% CI: 85–98%). The diagnostic values of a range of troponin levels for predicting inpatient mortality is displayed in Table 4. As the troponin threshold reduced, there was an increase in sensitivity and NPV (Table 4). As the troponin threshold increased, there was an increase in specificity while PPV changed minimally (Table 4).

**Table 4:** Diagnostic parameters of hs-cTnT thresholds for inpatient mortality.

Hs-cTnT (ng/L)	Sensitivity	95% CI	Specificity	95% CI	PPV	95% CI	NPV	95% CI	+LR	95% CI	-LR	95% CI
3	100	93–100	5	2–10	28	21–35	100	59–100	1.05	1.0–1.1	0	-
5	100	93–100	13	8–20	30	23–37	100	81–100	1.15	1.1–1.2	0	-
10	94	84–99	39	31–48	36	28–45	95	86–99	1.55	1.3–1.8	0.15	0.05–0.5
14 (Normal)	92	81–98	45	37–54	38	29–47	94	85–98	1.68	1.4–2.0	0.17	0.07–0.5
20	86	74–94	55	46–63	41	32–51	92	84–97	1.92	1.5–2.4	0.25	0.1–0.5
23 (Youden)	84	71–93	61	53–69	44	34–55	91	84–96	2.19	1.7–2.8	0.26	0.1–0.5
25	78	65–89	64	56–72	44	34–55	89	81–94	2.20	1.7–2.9	0.34	0.2–0.6
30	69	54–81	67	59–75	43	32–55	86	78–92	2.09	1.5–2.8	0.47	0.3–0.7
35	65	50–78	68	59–76	42	31–54	84	76–90	2.01	1.5–2.8	0.52	0.4–0.8
40	59	44–72	72	64–79	44	32–56	83	75–89	2.11	1.5–3.0	0.57	0.4–0.8
45	57	42–71	74	66–81	45	32–58	83	75–89	2.21	1.5–3.2	0.58	0.4–0.8
50	45	31–60	79	71–85	43	30–58	80	72–86	2.10	1.4–3.3	0.70	0.5–0.9
55	41	28–56	79	71–85	41	28–56	79	71–85	1.92	1.2–3.0	0.75	0.6–1.0
60	41	28–56	81	74–88	45	30–60	79	72–86	2.22	1.4–3.6	0.72	0.6–0.9

CI: Confidence interval; hs-cTnT: High sensitivity cardiac troponin T; +LR: Positive likelihood ratio; -LR: Negative likelihood ratio; NPV: Negative predictive value; PPV: Positive predictive value. Normal denotes the hs-cTnT threshold derived from the 99^th^ percentile of healthy individuals widely used in clinical practice for detecting cardiac injury. Youden denotes the optimal threshold based on the receiver operating characteristics curve shown in Fig 3.

## Discussion

Whilst several studies have associated elevated high sensitivity cardiac troponins with an increased risk of mortality on a patient group level [8,10,11,31–33], the clinical translation of this important biomarker for guiding clinical decision-making in acute COVID-19 has yet to take place. We showed in this study cohort that having an elevated troponin level did not necessarily mean that the patient would suffer inpatient mortality. Such poor positive predictive value of an elevated troponin level for mortality hinders the clinical development of a reliable threshold for identifying high-risk patients. The second key finding is that a normal high sensitivity cardiac troponin excludes inpatient mortality with a respectable negative predictive value in this study cohort. This observation gives rise to the notion that cardiac troponins are better developed as clinical rule-out tests for mortality in order to identify low-risk COVID-19 patients.

This paper should perhaps lead to scientific and clinical thoughtfulness about whether cardiac troponins are better used as a rule out rather than a rule in measure when we are considering mortality. The aim is to move forward from the observed group-based link between troponins and mortality in the existing literature, to a more acute understanding of the diagnostic property of cardiac troponins in COVID-19 and how we could exploit it for clinical translation.

**Fig 4:**
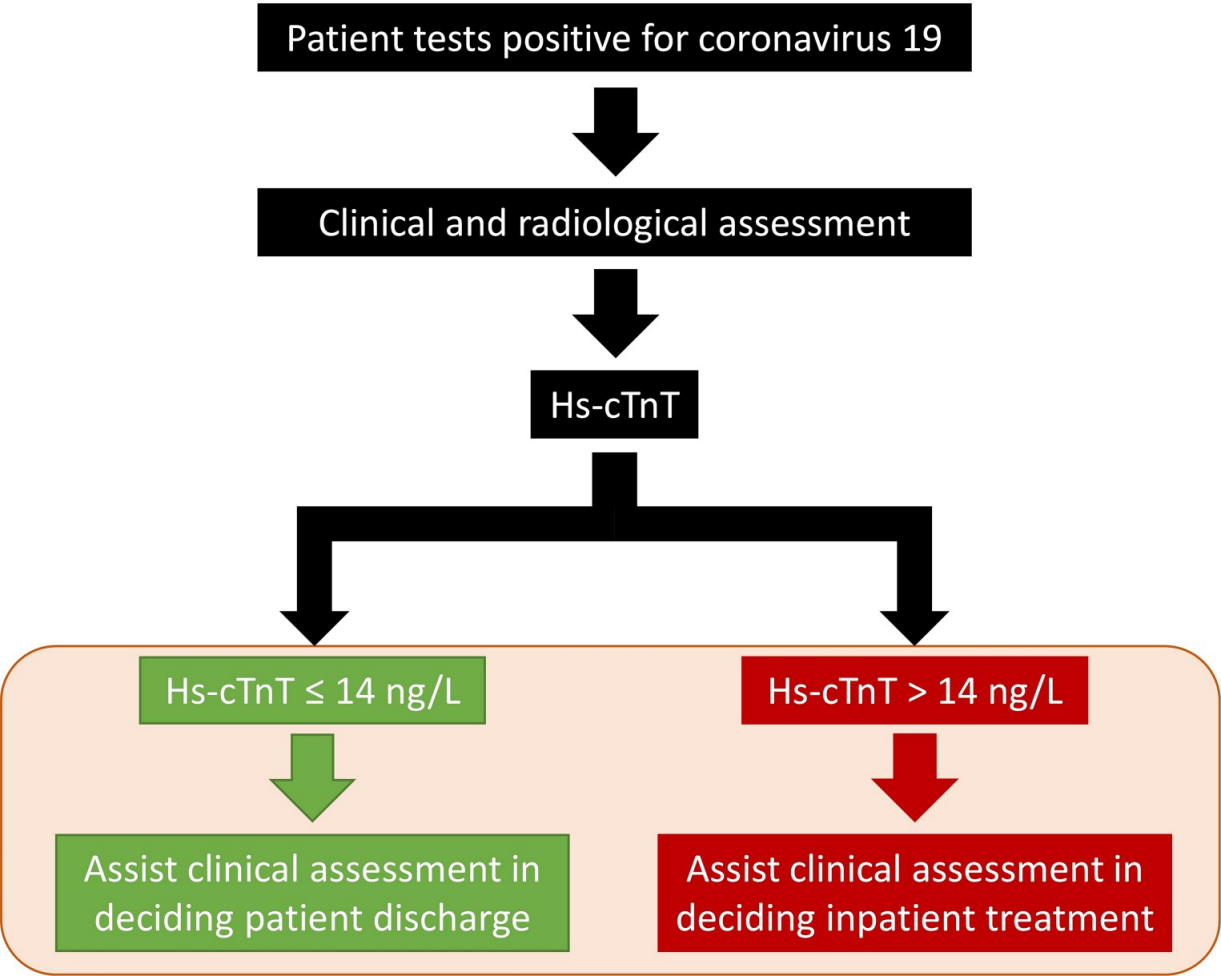
Future directions. Whether hs-cTnT can be used to assist clinical assessment to decide on patient discharge or inpatient treatment requires further investigation, as denoted by the orange panel. Hs-cTnT: High sensitivity cardiac troponin T.

As it stands, the results do not yet support the direct incorporation of cardiac troponins into clinical decision-making in COVID-19 patients. Instead, this study sets the stage for the prospective validation of a normal troponin as a rule-out test, to facilitate admission-avoidance and early safe discharge (Fig 4). These prospective data could provide the missing link in transitioning cardiac troponins and prognosis from an interesting observation in COVID-19 to a practical risk stratification tool in management pathways.

### High sensitivity cardiac troponin is a poor predictor of individual mortality risk in COVID-19

Our observation of higher overall mortality risk in the COVID-19 patient group with elevated high sensitivity cardiac troponin is supported by other studies [8,10,12,31]. However, this population-based trend did not translate to diagnostic usefulness in the individual patient. Only a small proportion of patient with an elevated troponin suffered mortality (~39.7%; Table 2), which led to poor positive predictive valves (PPV) on ROC analysis. Even at a relatively high troponin threshold of 60 ng/L, the PPV for mortality was only 45% (95% CI: 30–60%; Table 4). Consequently, an elevated troponin is an inaccurate predictor of mortality on an individual basis in COVID-19, hindering its incorporation into clinical decision-making or guidelines.

The failure of high sensitivity cardiac troponin to accurately predict mortality in COVID-19 likely rests on the fact that an elevated troponin in an unselected population is non-specific [34]. Indeed, an elevated troponin may be caused by a wide range of different pathologies [34], including both cardiac causes, e.g. hypertension, IHD, AF, heart failure, and non-cardiac causes, e.g. CKD, COPD and CVA/TIA. Each of these underlying pathologies can exert a different degree of detrimental effect on prognosis in COVID-19 [35–37]. Recent studies have shown that septic shock and multi-organ failure are the commonest causes of death in COVID-19 patients [38,39], including in an autopsy study [38]. Respiratory failure is a less common cause of death [38]. Cardiac troponin elevations can occur in most of the common causes of death in COVID-19 (such as septic shock and multi-organ failure) [40]. The non-specific nature of an elevated troponin will remain a major hindrance to any clinical pathway seeking to utilise it to predict mortality in COVID-19. This issue is further compounded by the fact that patients in real-life practice will always present with a range of co-morbidities, which cannot be controlled. Notwithstanding the link between elevated troponin and mortality, it is noteworthy that the potential use of a negative troponin to rule out mortality appears to be more promising for clinical translation.

In this study, patients with elevated vs normal troponin levels had similar rates of non-fatal complications, despite exhibiting significant differences in mortality rates. This observation appears similar to some other studies [20,41]. The cause of mortality in COVID-19 patients is often multi-factorial and remains incompletely understood [39]. Patients requiring NIV, intubation or ICU admissions do not necessarily suffer mortality [39]. Whilst a significant proportion of COVID-19 patients can develop respiratory failure requiring NIV/intubation, patients can also succumb to other causes such as sepsis, myocardial injury, multi-organ failure or secondary infection, which may not result in NIV/intubation or ICU requirement [39]. Further work is needed to elucidate the association between fatal and non-fatal complications of acute COVID-19.

### High sensitivity cardiac troponin is a potentially excellent rule out test in COVID-19

As a clinical test, high sensitivity cardiac troponin has a rapid turnaround time with results being available within an hour [42], which fits with most clinical decision timeframes [43]. The excellent negative predictive value of a normal troponin observed in our data is similar to other studies [8,10,11,19,31,33,44]. The results of this study strongly indicate that the true clinical utility of troponin in COVID-19 may rest on it being a good rule-out test on an individual patient diagnostic level. A negative troponin would eliminate the influence of acute cardiac pathologies and the heterogeneous effects of confounders on prognosis. As a biomarker, the high sensitivity cardiac troponin threshold of 14 ng/L used in this study was derived from the 99^th^ percentile of a healthy reference population [22]. Although the ROC analysis identified a troponin threshold of 23 ng/L as the Youden point, this threshold would not provide optimal clinical utility, since it does not provide the best NPV to rule-out inpatient mortality, which the 14 ng/L threshold enables (Table 4).

### Strengths and limitations of this study

This is a retrospective single-centred study and the results cannot yet be directly applied in clinical practice. Although troponin assays were ordered at the discretion of the treating physician at the time, owing to the paucity of data on the use of troponins in COVID-19, the assay results would not have been systematically used to guide clinical decision making. Instead, this study provides a blueprint for the further validation of cardiac troponins in clinical practice. This study is also limited by potential selection bias based on troponin measurement, since the measurement of cardiac markers is not routinely performed in COVID-19 patients. Therefore the conclusion of the study applies to this cohort of patients and again requires further prospective validation. In this study, high sensitivity cardiac troponin was measured on a single time point and were repeated in some but not all patients. This is understandable given that during the study period, the use of troponins in COVID-19 was not established nor evidence-based. The lack of repeat measurements meant that we could not differentiate between acute cardiac injury (rise and fall in troponin) [45] and chronic troponin elevations [46]. Moreover, this patient cohort originated from the pre-vaccination era and further studies are needed to elucidate the diagnostic value of troponins in vaccinated populations. Finally, treatment strategies for COVID-19 were not standardised during the study period. For example, patients were frequently offered nasal high-flow oxygen therapy in the early pandemic [47], while the practice switched to a preference for continuous positive airway pressure (CPAP) ventilation later on [48]. Further work is needed to study the effect of emerging and novel therapies on the prognostic value of high sensitivity cardiac troponin.

## Conclusion

In this study cohort of COVID-19 patients, the potential clinical utility of hs-cTnT appears to rest in ruling out inpatient mortality. This finding, if prospectively validated in a larger study, may allow hs-cTnT to become an important biomarker to facilitate admission-avoidance and early safe discharge.
